# A Research Agenda for Malaria Eradication: Cross-Cutting Issues for Eradication

**DOI:** 10.1371/journal.pmed.1000404

**Published:** 2011-01-25

**Authors:** The malERA Consultative Group on Integration Strategies

## Abstract

Discipline-specific Malaria Eradication Research Agenda (malERA) Consultative Groups have recognized several cross-cutting issues that must be addressed to prevent repetition of some of the mistakes of past malaria elimination campaigns in future programs. Integrated research is required to develop a decision-making framework for the switch from malaria control to elimination. Similarly, a strong economic case is needed for the very long-term financial support that is essential for elimination. Another cross-cutting priority is the development of improved measures of intensity of transmission, especially at low and nonrandom levels. Because sustained malaria elimination is dependent on a functioning health system, a further key cross-cutting research question is to determine how inputs for malaria can strengthen health systems, information systems, and overall health outcomes. Implementation of elimination programs must also be accompanied by capacity building and training to allow the assessment of the impact of new combinations of interventions, new roles for different individuals, and the operational research that is needed to facilitate program expansion. Finally, because community engagement, knowledge management, communication, political, and multisectoral support are critical but poorly understood success factors for malaria elimination, integrated research into these issues is vital.

## Abstract 2

The Malaria Eradication (malERA) Consultative Group on Cross-Cutting Issues draw out common themes identified by different discussion groups that must be addressed to prepare for malaria eradication.

**Summary Points**

- Several important cross-cutting issues must be addressed as the international community or an individual program moves from malaria control to malaria elimination/eradication: an integrated decision-making framework must be constructed for this paradigm shift
- Methods to measure transmission rapidly and cost-effectively in populations, particularly in low transmission settings, must be developed; very sensitive indicators of transmission are particularly important late in the elimination phase
- Elimination programs must be integrated for mutual benefit with strengthened health systems; better training and capacity building, better information systems, and modeling must also be developed
- New or improved tools alone will not be enough; community engagement and good communication between everyone involved in malaria elimination/eradication is essential
- A research and development agenda for cross-cutting issues is presented that should facilitate progress as programs aiming at malaria elimination/eradication supersede malaria control programs

## Introduction

During their deliberations, scientists in the various Consultative Groups contributing to the Malaria Eradication Research Agenda (malERA) concentrated on research questions relevant to their thematic areas. But, in addition, they also briefly noted many issues of relevance beyond their own domains. Some of these issues are likely to be critically important in malaria elimination/eradication programs. Consequently, they received special attention from the malERA Consultative Group on Integration Strategies. In this paper, we focus on the research and development needs of these important cross-cutting issues, especially in the context of historical reports of reasons for the failure of past campaigns. Consideration of these cross-cutting issues, we argue, is essential for regional elimination and, ultimately, global eradication of malaria, but is also relevant for scaled-up and improved control of disease.

## The Historical Context

The Consultative Group identified many cross-cutting topics of special significance by examining reports of the failures and successes of earlier approaches to regional elimination of malaria. History reveals that political, social and human factors are likely to be just as important as, if not more important than, biological and technological factors, and that a multidisciplinary approach to elimination/eradication is essential. Accordingly, special attention was given during the malERA consultations to finding synergies and strategies to prevent the “silo effects” that can occur when specialist groups work in isolation. It is important to identify critical partnerships between malaria elimination/eradication programs and programs in health or education, such as integrated management of childhood illness. Similarly, it is important to recognise the need to address social determinants of health for successful malaria eradication campaigns. Finally, ongoing critical analysis of the success or failure of current elimination efforts constitutes a research agenda in its own right, as exemplified in numerous campaigns against other diseases [1],[2].

## The Global Malaria Action Plan and Research for Eradication

The Global Malaria Action Plan (GMAP) [3] is focused predominantly on control, but nevertheless includes eradication as an ultimate goal. The malERA process, with its paradigm shift from control to elimination, has produced significant additions to GMAP by defining a research agenda that will assist in interruption of transmission. The malERA process emphasises the importance of clearly defining the essential research and development needed to achieve specific goals. That is, it focuses on the minimal essentials—what we “need to know”—rather than what would be maximally possible to know or even “nice to know.”

## Research for Readiness to Attempt Regional or National Elimination

The GMAP has identified the need to continue and scale up control of malaria in highly endemic areas for maximal reduction of morbidity and mortality, and recognises this as a priority for the foreseeable future [3]. As the malaria map shrinks and malaria incidence falls, some countries may consider attacking remaining foci with an elimination agenda. Many pre-elimination considerations are related directly to the competence and readiness of the health system, and are discussed in the malERA paper on health systems and operational research [4]. Decision-makers must also balance the consequences of diverting resources from urgent clinical needs to a problem that by definition is causing little morbidity.

Importantly, decision makers at national and regional levels may need to be reminded that successful elimination for a few years will inevitably lead to loss of the naturally acquired immunity that is a good defence against malaria. Attempts to eliminate malaria that are not sustained can therefore provide the grounds for serious epidemics in people of all ages, with rapid loss of the gains accrued during an elimination program if the program fails or is stopped prematurely [2],[5].

The Malaria Elimination Group has recently highlighted the immediate needs of governments that are currently facing important decisions about malaria elimination/eradication [6]. Political commitment is essential; local research agendas for drug and insecticide resistance must be completed, health system readiness assessed, and cost-effectiveness analyses undertaken before deciding to make the long-term investment in elimination.

From its discussions, the malERA Consultative Group on Integration concluded that the cross-cutting research and development agenda in the context of the paradigm shift from control to eradication must take into account the research developments of the last few decades. Since the end of the Global Malaria Eradication Program (GMEP), innovations such as rapid diagnostic tests, insecticide-treated bednets, and improved information systems and communication systems have been developed, and a partially effective vaccine should be available in the foreseeable future. Thus, an algorithm needs to be defined and a tool developed for deciding the readiness of the system for elimination, or even for introduction of one of these innovations.

## The Case for Long-Term Investment for Eradication

Cross-cutting research is needed to make the case for long-term investment in eradication for the global public good and to ensure that financial support is available for the “last mile” before elimination [7]. This case should align with, and complement, important and related development themes such as global security, migration, food security, and climate change. If research findings suggest that the case is strong, malaria eradication could be included in global policies for health that follow on from the Millennium Development Goals beyond 2015 [8]. Importantly, a development agenda consistent with the Paris Declaration on Aid Effectiveness and the Accra Agenda for Action [9],[10] should be accompanied by strong harmonization with the GMAP and the goals of the Roll Back Malaria Partnership [3].

## Cross-Cutting Research for a Good Measure of Transmission in the Later Stages of Elimination

Malaria elimination has a very different endpoint from malaria control and this change of paradigm demands the development of specific measures of progress. New infections are a direct measure of ongoing transmission but require labor-intensive, active surveillance studies, particularly during the elimination phase in regions previously experiencing high transmission where immune individuals are unlikely to experience symptomatic disease. After some years, as immunity declines, infection is more likely to be symptomatic and may then be a good surrogate marker for the detection of continued or resumed transmission during surveillance. Thus, at the end of the process, some years after elimination has been achieved and the population has lost clinical immunity, surveillance of clinical cases can become a guide to transmission. However, there are many years between the time when transmission can be measured in endemic areas (albeit with difficulty and high cost) and the time when active surveillance of occasional cases becomes a useful measure (see also [11],[12]).

Accordingly, elimination programs need rapid, sensitive, standardised, and reproducible transmission measurement methods to monitor progress towards the desired goal [13], particularly when transmission continues at low and nonrandom levels. Research into and development of new measures that are simpler than surveillance for incident infections is a high priority in the cross-cutting research and development agenda. Such measures could potentially be based on serological or other biomarkers and used as indicators of readiness for elimination, progress towards that goal, and as markers of residual foci or reintroduced infection [12].

In particular, the new and improved measures of transmission could be used for measurement and certification of the absence of transmission. Such measures are essential to ensure that the decision to stop expensive entomological studies or indoor spraying that inconvenience communities is made at the appropriate time. Sustained funding is, of course, required to detect ongoing transmission or reintroduction of disease.

## Integration with Strengthened Health Systems

Many past efforts at malaria elimination have failed because the health system failed during the implementation of stand-alone programs [2]. This failure, through neglect or at least under-resourcing during implementation of vertical programs, resulted in the pessimistic view that malaria can only be eliminated in regions where economic progress and stable governance are in place that support well-functioning health systems. Even if a region initially opts for a purely vertical approach, when transmission declines, patient needs for appropriate diagnosis and treatment in the general health system become part of the surveillance system and need to be integrated with existing health system structures for local responses and central monitoring [2],[4]. Moreover, diagnosis and appropriate treatment can contribute to reduction in transmission, and good health facilities are essential for management of other febrile illnesses. For these reasons, a malaria elimination program simply cannot succeed in the absence of an effective health system.

The importance of health systems thinking, the need for setting-specific and phase-specific integration, and the need for new approaches to replace the old separation into “horizontal” or “vertical” programs have been discussed by most of the other malERA Consultative Groups but particularly by the group that focused on health systems [4]. The consultative groups also highlighted relevant cross-cutting research and development agenda topics such as the need to measure synergies between malaria-specific programs and health systems strengthening, and the extent to which inputs for malaria elimination can be used to strengthen population health. Our group concluded that tailoring an approach to each setting is required, maximising synergy with the health system for mutual benefit, while maintaining the integrity of categorical program objectives, and the important activities of the health system.

## Training

All of the consultative groups recognized the need for training and capacity building in the context of elimination, from discovery research in the laboratory, through social sciences research in communities, and on to operational research in the context of health systems thinking. Master's level research training that introduces the principles of a scientific approach, epidemiology, and evidence-based decision making would benefit anyone involved in deciding about resource allocation, timing, and refinement of the elimination approach before, during, and after any elimination/eradication program. Training for the eradication research agenda also needs to be accompanied by training of public health leaders and managers with substantial knowledge of malaria.

In addition, communities of health systems experts require research training to help them measure the impacts of an integrated approach to malaria elimination. “Elimination science” would assess the implementation of changed diagnostic or surveillance methods, or expanded roles of community health workers or reporters engaged in active surveillance (“learning in action”). The information gleaned through such assessments could be used for operational research or social science research relevant to community participation and engagement. It could also be used by a new cohort of experts in database development, management, or information technology.

For basic research, which has a longer time frame, academic expertise needs to be developed and sustained in fields relevant to technological development such as bioinformatics, genetics, drug and vaccine discovery, systems thinking, and mathematical modeling. It also needs to be developed in fields relevant to health promotion and communication and the enhancement of these fields by new technology.

Together, these training requirements, particularly those that focus on the needs of disease-endemic countries, are substantial and should be the subject of a later specific review.

## Information Systems and Modeling for Assessing Combinations of Intervention Strategies

All the consultative groups acknowledged the importance of strong information systems that are reliable and responsive to local needs for rapid intervention, and that provide inputs to national and regional databases. The requirements for information systems will change over time with changes in transmission but an important attribute of these systems should be harmonization and the avoidance of unnecessary duplication to meet, for example, special or frequent requests from funding agencies. Importantly, additional sources of information have to be integrated into existing information systems to allow modeling of future interventions, to facilitate the analysis of system-wide effects for costing and implementation, and to provide a resource for researchers who are modeling transmission, as discussed in other malERA articles (also see [4],[14]).

In common with surveillance systems, information systems need to be envisaged as tools for intervention (with a target product profile and standards to be developed and monitored), rather than as ends in themselves. The consideration of information systems as interventions (just as surveillance was defined as an intervention by the WHO Global Malaria Eradication Program), provides a useful perspective for the definition of the malERA research and development agenda and is well discussed elsewhere in this series.

Finally, because the costs and benefits, potential synergies, and operational assessments of combination strategies are likely to be different in different environments, modeling emerged as one of the key cross-cutting themes during the malERA consultation process. In particular, the use of modeling to assist discussions and decisions on intervention mixes in time and space emerged as a high priority cross-cutting theme that is discussed further in the relevant article in this Supplement [14].

## Community Engagement

Successful public health programs are characterized by community engagement and good communication, but how to achieve these critical success factors is not well understood. Community case management and treatments such as piloted in Tigray [15], can be effective, but support from all sectors of society is critical, particularly where there is a requirement for behavioural change. Strategies are required to explain why efforts against malaria need to be maintained, even when malaria cases are extremely rare. Conversely, governments also have to choose the correct time, and explain the rationale for stopping certain interventions. We need to understand how public perception affects such decisions and provide guidance for countries on when certain interventions will no longer be cost-effective, and we have to communicate this information effectively.

Good communication is essential among malaria researchers. It is also essential that malaria researchers communicate well with people involved in health systems, malaria control specialists, health care workers, funders, stakeholders from public and private nongovernment sectors, communities, the general population, and the international community. Research should be undertaken on the range of factors that influence connectivity, from cultural aspects to technology, which could be revolutionised by the advent and availability of new means of communication.

## Conclusions

An important part of the malERA process was to identify cross-cutting issues that could facilitate the achievement of the goal of elimination, particularly in the light of past failures, and build on the GMAP that already includes eradication as a long-term goal. As recognized by the whole malaria community, integration is a prerequisite for success.

Tools alone are not enough, but need to be accompanied by excellent and ongoing coordination, operational research, information systems, and monitoring and evaluation supplemented by active surveillance. Integration with the health system and a multidisciplinary approach are also essential, providing new tools and approaches for modeling and for systems thinking about the concepts and strategy needed to achieve the ultimate goal. In addition, communication and research into its improvement and local adaptation are critical; without excellent communication and community and political engagement, elimination/eradication programs will not succeed. Moreover the community and the health system need to be ready with appropriate tools and trained personnel in place to take on new or specific tasks that need to be integrated into ongoing activities.

Before attempting elimination, a realistic feasibility assessment is required to determine readiness for this challenge. Some countries fall far short of readiness, having tools that are inadequate to complete the task where force of infection is very high, having health systems that are weak, or suffering from socio-political and civil disturbances that make public health practice nearly impossible. Other countries may simply lack one major prerequisite such as political will, or a drug to overcome resistance to available antimalarial therapy. Unrealistic promises about malaria elimination will inevitably lead to disappointment and disillusion with public health approaches and should be avoided.

We cannot provide estimates of the cost of the research and development agenda for cross-cutting issues that we present in Box 1, and recognise that further work will be required to delineate fully all the regulatory and ethical implications of new tools that have been envisaged or described here. Technology that may provide solutions may currently be beyond our imagination, but could be available within a short few years. Importantly, however, we recognize that very long-term investments will be needed for the research and development agenda that we have outlined. We also recognize that we need to build on public/private partnerships and connections with industry to facilitate new advances. Nevertheless, we emphasize that, even if elimination programs are decades away for some countries with very high transmission, now is the time to start work on the broad and integrated portfolio of long-term research that is essential if the goal of malaria eradication is to be achieved.

**Box 1. Summary of the Research and Development Agenda for Cross-Cutting Issues**

- Develop and validate a framework of essential information required for making the decision to progress from scaled-up control to elimination that includes political, economic, and financial factors. The framework should recognise variability in epidemiology, the need for political will to prioritise and/or finance and support such a long-term project, and the need for locally effective tools powerful enough to finish the task.
- Develop a long-term investment case for elimination that should align with important development themes such as global security, migration, food security, and climate change.
- Document current and past efforts towards elimination.
- Develop methods and approaches to measure and monitor transmission in a rapid and cost-effective way at a population level, especially in very low transmission settings. These methods and approaches should be used as metrics for the very sensitive indicators of progress needed for active surveillance systems required in the last phases of elimination.
- Define and develop the tools required for a communication and knowledge management strategy that encourages community engagement, local health system involvement, and the participation of national, and international stakeholders.

## Abbreviations

GMAP: Global Malaria Action Plan
